# Sugar feeding in triatomines: a new perspective for controlling the transmission of Chagas disease

**DOI:** 10.3389/fphys.2024.1360255

**Published:** 2024-10-15

**Authors:** Mariana C. Costa, Carlos J. C. Moreira, Pedro Lagerblad de Oliveira, José Juberg, Daniele Pereira de Castro, Fernando Ariel Genta

**Affiliations:** ^1^ Laboratório de Bioquímica e Fisiologia de Insetos, Instituto Oswaldo Cruz, Fundação Oswaldo Cruz, Rio de Janeiro, Brazil; ^2^ Laboratório de Doenças Parasitárias, Instituto Oswaldo Cruz, Fundação Oswaldo Cruz, Rio de Janeiro, Brazil; ^3^ Laboratório de Bioquímica de Artrópodes Hematófagos, Centro de Ciências da Saúde, Universidade Federal do Rio de Janeiro, Rio de Janeiro, Brazil; ^4^ Instituto Nacional de Ciência e Tecnologia em Entomologia Molecular, Rio de Janeiro, Brazil; ^5^ Laboratório Nacional e Internacional de Referência em Taxonomia de Triatomíneos, Instituto Oswaldo Cruz, Fundação Oswaldo Cruz, Rio de Janeiro, Brazil

**Keywords:** triatomine, sugar feeding, Chagas disease, attractive toxic sugar bait (ATSB), *Rhodnius prolixus*

## Abstract

**Introduction:** Triatomines are vectors of *Trypanosoma cruzi*, the etiological agent of Chagas disease. Currently, there is no vaccine against this disease. Thus, control of the insect vector population is the main strategy available to reduce the number of cases. Triatomines are considered obligate hematophagous, but different alternative feeding behaviors were described, such as haemolymphagy or plant feeding.

**Methods:** To determine the preference for sugar feeding in nymphs and adults of *Rhodnius prolixus*, the insects were exposed a piece of cotton containing bromophenol blue plus sucrose. In addition, we offered several sugars for different species of triatomines, and tested sugar meals as a route of delivery of insecticides in first-instar nymphs of *R. prolixus*. The effect of sugar feeding on the physiology of these different species of triatomines was recorded.

**Results:** First instar nymphs ingested sucrose more strongly than other stages, and showed high mortality rates. In different species of triatomines, sucrose induced an ingestion, but engorgement varied according to the species. *R. prolixus* nymphs showed an indiscriminate intake of various sugars, with very different physiological effects. Furthermore, ingesting different combinations of insecticides + sugar significantly reduced insect survival.

**Discussion:** In summary, we described for the first-time sugar feeding as a widespread behavior in several species of triatomines, and the possibility of the use of toxic sugar baits for the control of these vectors. The knowledge of feeding behavior in these insects can be fundamental for the development of new strategies to control Chagas disease.

## 1 Introduction

Triatomines (Triatominae, Hemiptera, Reduviidae), popularly known as kissing bugs, are insect vectors of the flagellated protozoan, *Trypanosoma cruzi* (Protozoa, Sarcomastigophora, Kinetoplastida, Trypanosomatidae) (Chagas, 1909), which causes Chagas disease or American Trypanosomiasis. It is a severe and fatal pathology, endemic in the American continent and until now there is no vaccine or definitive cure for this illness. The World Health Organization estimated that there are about 6-7 million infected people in the world, causing approximately 10,000 deaths per year (World Health Organization, 2022). There is a huge concern that with global warming and migration of human populations, new cases of the disease can occur in the US, Europe or even Asia, with the spreading of insect vectors and the stablishment of new cycles for the parasite (World Health Organization, 2022).

Transmission can occur through contact with the feces and urine of infected triatomines, in the bite area, mucous membranes at the lips or in the eye regions, as well as blood transfusion, organ transplantation, congenitally (mother to child) or oral route (Blanchet et al., 2014; Coura, 2015). In recent years, oral transmission was recorded with greater frequency in some countries in South America, mainly in northern Brazil (Pará, Amapá and Amazonas), Colombia, Venezuela, Bolivia, and French Guiana (Alarcón de Noya et al., 2010; Santalla et al., 2011; Blanchet et al., 2014; Soto et al., 2014; Vargas et al., 2018). This type of transmission occured through the ingestion of contaminated material with parts of the infected triatomines and/or their feces. The different recorded outbreaks were attributed to the ingestion of contaminated foods, such as açaí berries, guava juice, sugarcane juice, açaí juice, bacaba juice, and contaminated water (Dias et al., 2008; Nóbrega et al., 2009; Alarcón de Noya et al., 2010; Blanchet et al., 2014; Vargas et al., 2018; Santana et al., 2019).

Currently, there are more than 150 species of triatomines in nature (Oliveira and Alevi, 2017; de Oliveira et al., 2018; Dorn et al., 2018; Lima-Cordón et al., 2019) and the genera *Panstrongylus*, *Rhodnius* and *Triatoma* are considered the main vectors of medical importance. These insects colonize several habitats, peridomicile (chicken coops, pens and stables), intradomicile and wild environments (palm trees, rocks, trunks, tree tops, animal nests, and bromeliads) (Jurberg et al., 2014). Although these insects were characterized by the obligate hematophagic feeding habit (Lehane, 2005), triatomines had alternative feedings, such as hemolymphagia, “cleptohematophagy” (cannibalism), coprophagy and/or feeding from sugary sources (Ryckman, 1951; Lorosa et al., 2000; Sandoval et al., 2000; Ruas-Neto et al., 2001; Díaz-Albiter et al., 2016).

Recent studies observed for the first time that first instar nymphs of *Rhodnius prolixus* ingested *Solanum lycopersicum* (cherry tomato), which resulted in several physiological gains for the insect such as reduced weight loss caused by desiccation, increased life expectancy and increased intake of blood (Díaz-Albiter et al., 2016). Furthermore, all instars of *R. prolixus* ingested 10% sucrose under laboratory conditions (Díaz-Albiter et al., 2016). Additionally, *R. prolixus* and *Panstrongylus geniculatus* adults ingested a drop of water, and adults of both species and fifth instar nymphs of *R. prolixus* also guava juice (Páez-Rondón et al., 2018).

Carbohydrates are organic molecules that have different functions in organisms, the most important is the supply of energy (Nelson and Cox, 2014). In nature, sap from plants, leaves, fruits and floral nectars are sources of sugar for various insects, and the composition and concentration vary between species (Nicolson and Thornburg, 2007). The nectar is basically composed of sucrose, glucose and fructose, but a wide variety of sugars have also been reported in different studies (Baker, 1982; Nicolson and Thornburg, 2007; Antoń et al., 2017), including xylose, raffinose, maltose, mellibiose, among others (Wykes, 1952; Percival, 1961; Van Wyk and Nicolson, 1995; Nicolson and Van Wyk, 1998).

It is important to point out that most adult insects, in Diptera for example, both males and females, need carbohydrates that are acquired in a daily basis directly from plants (in nectar or sap), aphid secretions, and ripe fruits (Cameron et al., 1995). In general, these sources of sugars are important for mating, survival, oviposition, as a general source of energy, also affecting the development and infectivity of intestinal parasites (Williams, 1970; Lewis and Domoney, 1996; Brazil and Brazil, 2003; Bates, 2007).

Because there is no vaccine available for Chagas disease, the control of the vector insect populations is the main strategy available to reduce the number of cases. Across Latin America, the basic strategy is the chemical control through the use of residual action insecticides, especially pyrethroids. However, continuous use of these compounds can generate resistance in triatomine populations (Zerba, 1999; Vassena et al., 2000; Picollo et al., 2005; Echeverria et al., 2018), making it necessary to develop other control strategies. Within this context, several known methods are not directly applicable to the control of triatomines and, therefore, the discovery of a new methodology, effective, low cost and applicable in the field, such as the elaboration of sugar baits, for example, may be essential to reduce the impact of this disease.

Toxic sugar baits were widely studied and had been successfully tested on vector insects, mainly in Diptera, as an insecticide delivery strategy (Müller and Schlein, 2008; Müller et al., 2010; Qualls et al., 2015; Gu et al., 2020). This method was implemented in the field or in residential environments, being harmless to humans due to its low toxicity. Sugar acted as a phagostimulant, caused the insect to ingest the toxic solution, which ended up dying. Therefore, sugar baits pointed to new paths for the development of vector control, capable of altering the development of the insect and effectively reducing the transmission of various diseases. In a broader context, the association of this type of method can be a viable future alternative for the control of triatomines, which can be effectively applied in the field, for being a simple and economical strategy. Although many aspects related to the sugar feeding in kissing bugs are still unknown, these new vector control actions deserve more detailed investigations.

The purpose of this work was to investigate whether different species of triatomines were capable of ingesting sugar solutions in the laboratory, characterize the physiological effects in insects fed with different types of sugars, as well as, test sugar meals as a route for the delivery of insecticides in first-instar nymphs of *R. prolixus*. In summary, we described here the first report of ingestion of sugar solutions in several species of triatomines, which had been considered exclusive hematophagous, and we showed that 1st instar nymphs of *R. prolixus* ingested different combinations of insecticides with the sugar, that significantly reduced the insect survival. These facts lead to the conclusion that the description of new food sources opened new perspectives for the development of new control strategies against triatomines.

## 2 Methods and scope of experiments

### 2.1 Insects maintenance

*R. prolixus* insects were obtained from an insectary at Laboratório de Bioquímica e Fisiologia de Insetos of Instituto Oswaldo Cruz at the Fundação Oswaldo Cruz. The triatomines fed defibrinated rabbit blood with the aid of an artificial apparatus (Azambuja and Garcia, 1997). The colony was bread at 28° ± 2°C and 60% ± 5% relative humidity.

Eggs from *Tritoma vitticeps, T. infestans, T. rubrovaria, T. dimidiata, Panstrongylus megistus*, and *Rhodnius neglectus* were obtained from Laboratório de Referência Nacional e Internacional em Taxonomia de Triatomíneos and Laboratório de Doenças Parasitárias at Instituto Oswaldo Cruz, Fiocruz. According to a previous investigation (Diaz-Albiter et al., 2016), first instar nymphs were used 14 days after hatching from the eggs and weighed (Electronic balance BEL model M214Ai, accuracy 0.1 mg, Piracicaba, Brazil). Only in the first series, that compared the ingestion of sucrose by nymphs and adults of *R. prolixus*, the period of starvation was unknown, and the insects were not weighed. Always ten insects were kept separately in a 100 mL glass vial containing 0.2 g cotton, wetted with 2 mL of the respective solution, closed by netting and placed in the insectary. Only in the toxic baits assays the conditions in the vials differed, they were kept inside an incubator at same temperature and humidity. The experiments were made in three biological replicates.

### 2.2 Offer of sugars to triatomines

To analyze the preference of sucrose intake in nymphs and adults of *R. prolixus* the piece of cotton was wetted with 10% (w/v) commercial sucrose solution plus 0.5% (w/v) bromophenol blue. In the control groups the sucrose solution was replaced by ultrapure water. After 2 days of exposure, survival was recorded. Living insects were immobilized on ice and dissected (by separating the anterior and posterior midgut from the rest of the body) in 0.9% (w/v) physiological saline under the aid of a Luxeo 4Z stereomicroscope (Labomed, India). The intestines that turned visibly blue were counted.

To assess 1st instar *R. prolixus* nymphs feeding on the different sugar solutions, the following solutions were offered: maltose, cellobiose, trehalose, galactose, raffinose, and glucosamine at 10% (w/v); and xylose, starch, xylan, pectin, carboxymethylcellulose, and laminarin at 0.25% (w/v). Control group insects were exposed to a piece of cotton wetted with 2 mL of ultrapure water. After 2 days of exposure, the insects were weighed again and survival was recorded.

To determine whether different triatomine species ingest sucrose, five first-instar nymphs of *T. infestans, T. dimidiata, T. rubrovaria, T. vitticeps, P. megistus and R. neglectus* were offered sucrose and water (control) as in the comparison of nymphs and adults of *R. prolixus* (see above). After 2 days of exposure, the insects were weighed again, photographed (using a cell phone attached to a Luxeo 4Z stereomicroscope) and survival was recorded.

### 2.3 Effect of toxic sugar baits on *R. prolixus* survival

In this assay, we tested sugar baits as an insecticide delivery strategy for kissing bugs. Twenty first-instar nymphs of *R. prolixus* were exposed to a piece of cotton (0.3 mg) wetted in 300 µL of a 10% trehalose plus 3 µL of ethanol stock solution containing one of the following insecticides: Triflumuron 10 mM; Temephos 10 mM; Deltamethrin 5 mg/mL; Permethrin 20 mg/mL, and boric acid 1% (w/v). Separated groups of insects were exposed in the same conditions to insecticide solutions in water only. Control groups were exposed to 300 µL of ultrapure water, or 300 µL of 10% trehalose.

Therefore, a total of 12 groups were defined for the assays: (1) boric acid; (2) boric acid + trehalose; (3) temephos; (4) temephos + trehalose; (5) triflumuron; (6) triflumuron + trehalose; (7) deltamethrin; (8) deltamethrin + trehalose; (9) permethrin; (10) permethrin + trehalose; (11) water; (12) trehalose. Water and trehalose solution were used as negative and positive controls. All groups were weighed after 24 h of treatment with different compounds. The survival was evaluated daily for 4 days after exposure.

### 2.4 Statistical analysis

Statistical analyzes of all experiments were performed using the software GraphPad Prism version 9.0 for Windows, GraphPad Software, Boston, Massachusetts USA, www.graphpad.com. We evaluated the normality of data distribution using the D’Agostino & Pearson test. Data that had normal distributions were submitted to unpaired *t*—tests and data with non-normal distributions were analyzed with the non-parametric Mann–Whitney test. We used Fisher’s exact test to analyze the survival experiments (Zar, 2010). The longevity curves were compared using the Log-rank (Mantel-Cox) test (Rai et al., 2021). The results represented the mean and the standard error of the mean (SEM). Significance was considered with *p* < 0.05.

## 3 Results

### 3.1 Ingestion of sugars by triatomines

Sucrose was offered to groups of different stages of *R. prolixus*, all containing 10 insects. None of the adults but 76% ± 12% of first instar nymphs, 46% ± 3% of second, 60% ± 15% of third, 27% ± 27% of fourth and 3% ± 3% of fifth instar nymphs visibly engorged sucrose, indicated by the blue contents of the anterior midgut. The engorgement rates of fifth instar nymphs and adults differed significantly from those of first and third instar nymphs (Fisher's exact test, *p* < 0.05). None of the nymphs and adults ingested water. Considering the mortality rates, in the sucrose-offered groups, none of the fifth instar nymphs and adults died within the observation period of 2 days, but 60 ± 15, 73 ± 3, 76% ± 12% and 90% ± 10% of the groups of first, second, third and fourth instar nymphs died, respectively. No mortality in the water offered groups was observed. There was a positive Spearman correlation between the engorgement rates and mortalities [r (16) = 0.920, *p* = 6.558.10^−8^].

**FIGURE 1:**
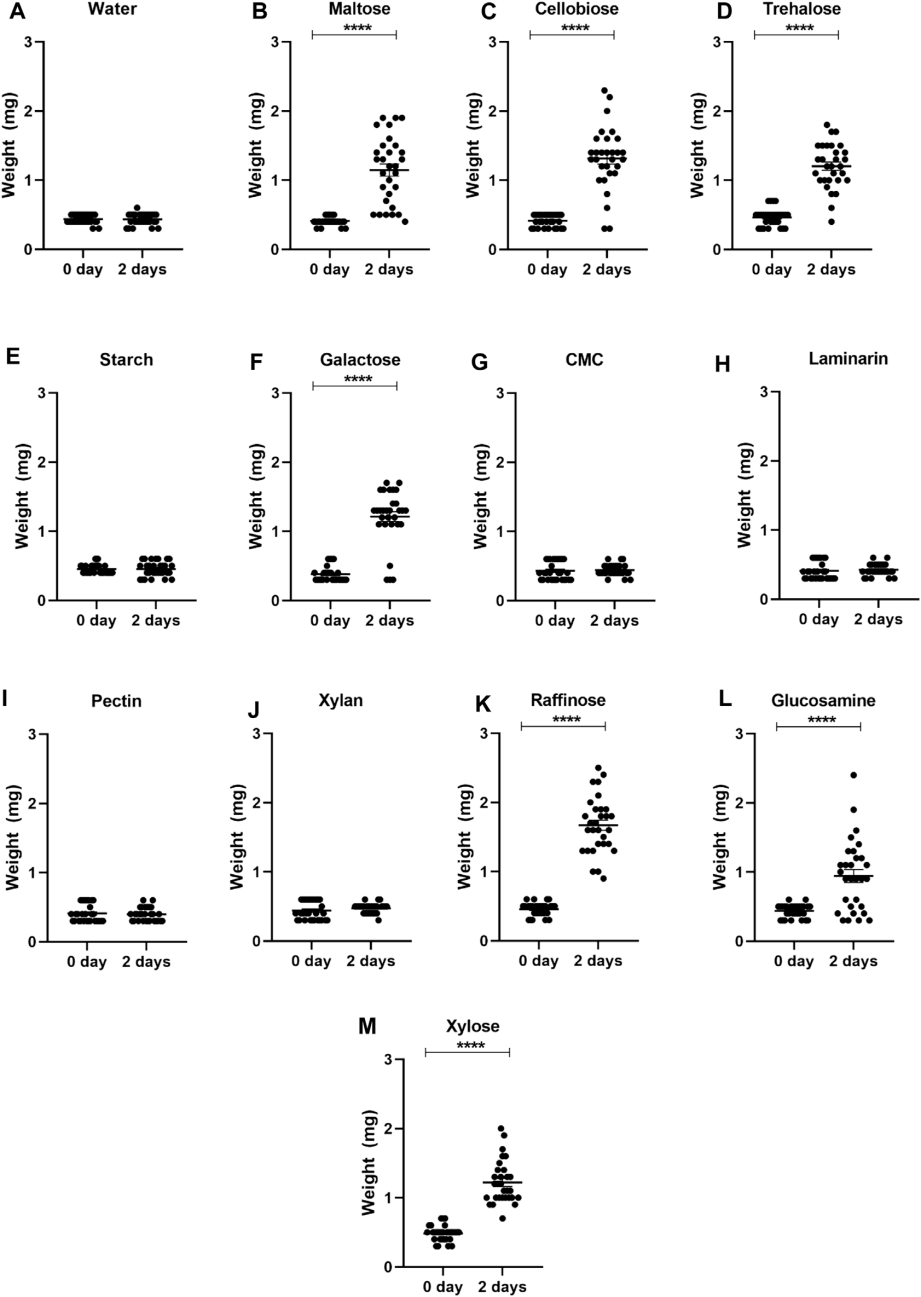
Weights of first instar nymphs of *Rhodnius prolixus* before (0 day) and after 2 days of exposure to water **(A)** or solutions of different sugars: maltose **(B)**, cellobiose **(C)**, trehalose **(D)**, starch **(E)**, galactose **(F)**, carboxymethylcellulose (CMC) **(G)**, laminarin **(H)**, pectin **(I)**, xylan **(J)**, rafinose **(K)**, glucosamine **(L)**, and xylose **(M)**. The values are mean and SEM obtained from groups of 10 insects. The experiment was repeated three times independently. Asterisks indicate significant differences compared to day 0 (*p* < 0.0001). Statistics: Unpaired t-test (water, maltose, trehalose, starafinosenose, glucosmine and xylose); Mann-Whitney test (cellobiose, galactose, CMC, laminarin, pectin and xylan).

**FIGURE 2:**
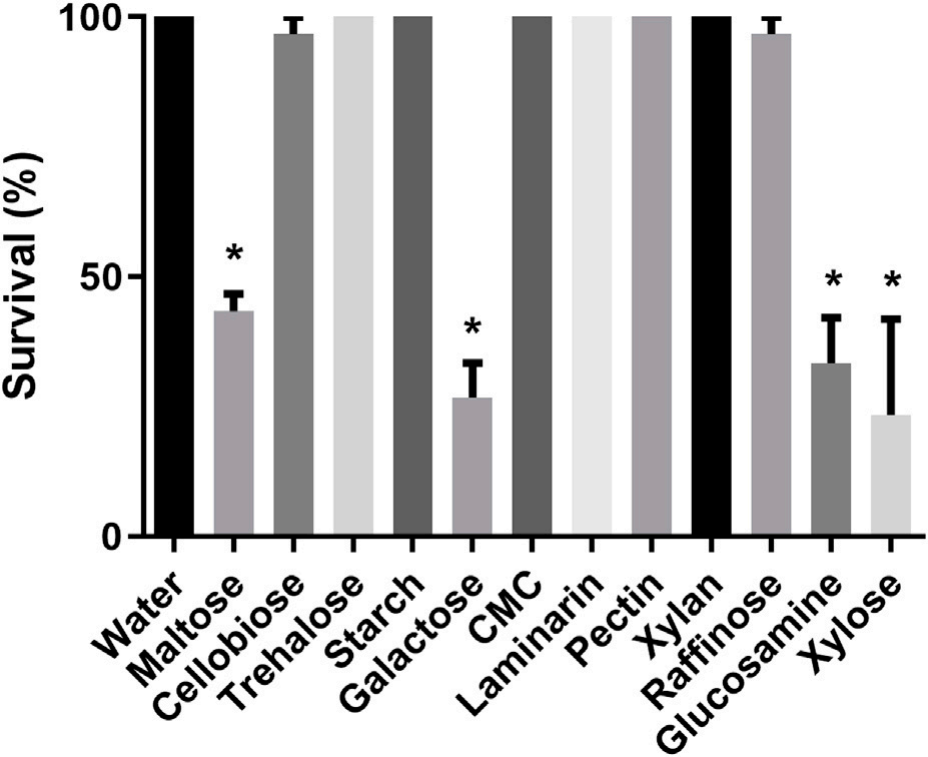
Survival of first instar nymphs of *Rhodnius prolixus* after 2 days of exposure to water or solutions of differents sugars: maltose, cellobiose, trehalose, starch, galactose, carboxymethylcellulose (CMC), laminarin, pectin, xylan, rafinose, glucosamine, and xylose. The values are mean and SEM obtained from groups of 10 insects. The experiment was repeated three times independently. Asterisks indicates statistical difference compared to water controls. (Fisher’s exact test), *p* < 0.05.

After observing the sucrose ingestion in nymphs and adults, we decided to assess if *R. prolixus* nymphs would ingest other types of sugars. Insects exposed to maltose, cellobiose, trehalose, galactose, raffinose, glucosamine, and xylose exhibited a significant increase in weight after 2 days of exposure (Figure 1), indicating an ingestion of the sugars. In contrast, insects exposed to starch, carboxymethylcellulose, laminarin, pectin, and xylan did not show weight gain. Again, control nymphs did not ingest water. Considering survival rates, all first instar nymphs that ingested trehalose survived and nearly all after ingestion of cellobiose and raffinose (Figure 2). Maltose, galactose, glycosamine and xylose killed more than 40%. Since starch, carboxymethylcellulose, laminarin, pectin and xylan had not been ingested, they did not affect the insects.

**FIGURE 3:**
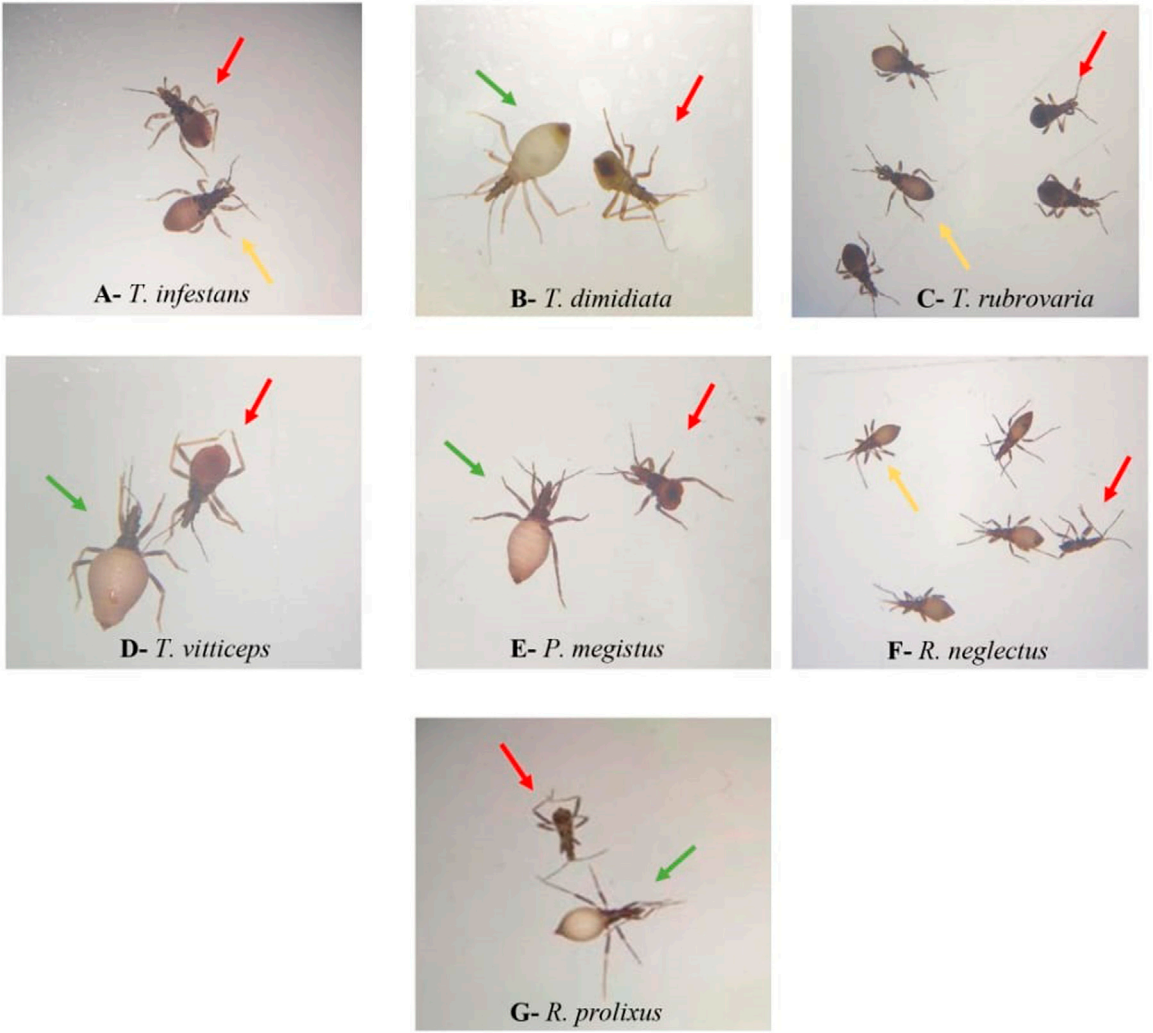
First instar nymphs of different triatomine species fully (green arrows) or partially (yellow arrows) engorged, next to visibly non-engorged insects (red arrows) after 2 days of exposure to sucrose 10%. *Triatoma infestans* **(A)**, *Triatoma dimidiata* **(B)**, *Triatoma rubrovaria* **(C)**, *Triatoma vittticeps* **(D)**, *Panstrongylus megistus* **(E)**, *Rhodnius neglectus* **(F)**, and *Rhodnius prolixus* **(G)**.

**FIGURE 4:**
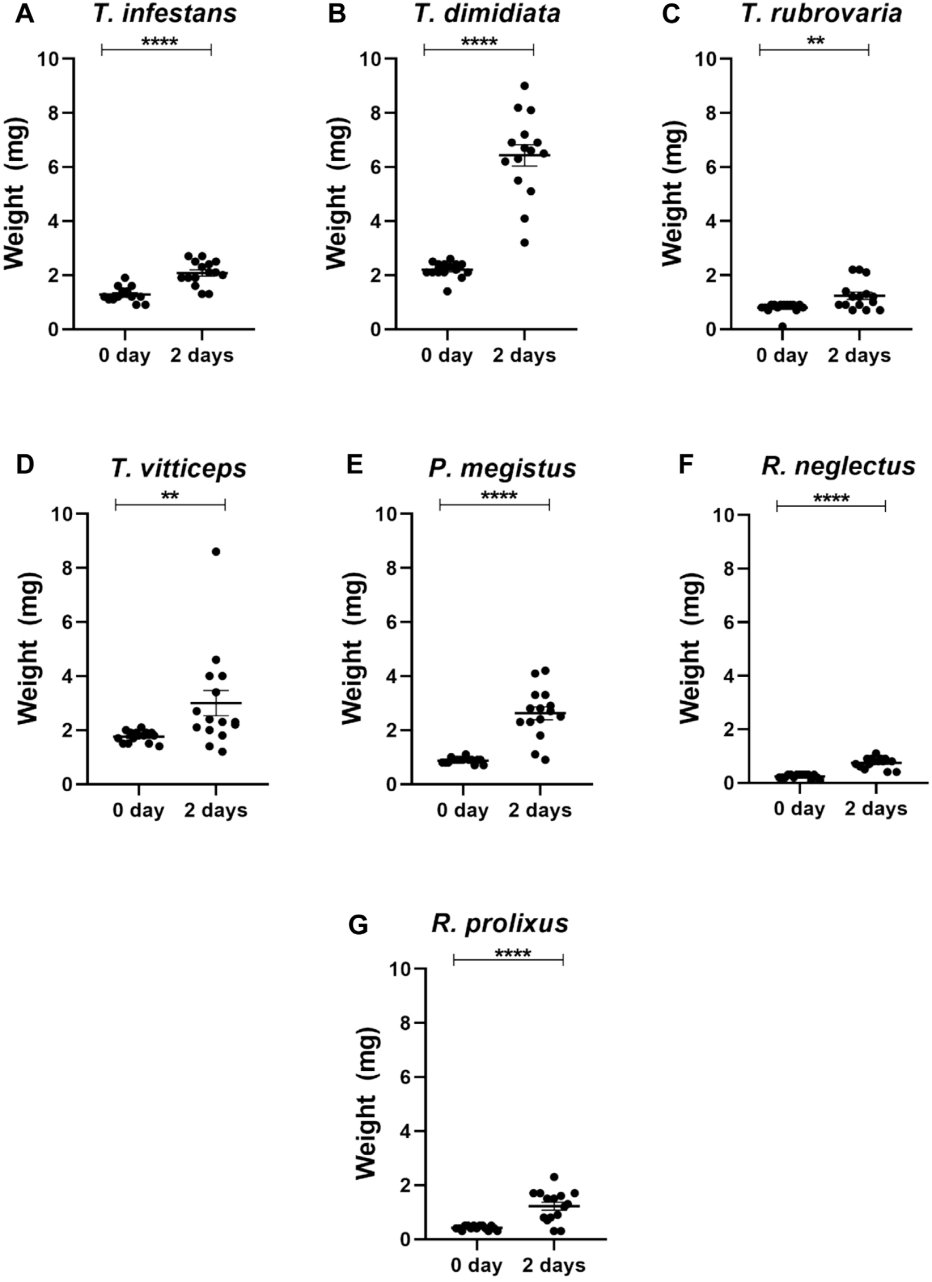
Weights of first instar nymphs of different triatomine species before (0 day) and after 2 days exposition to sucrose baits: *Triatoma infestans* **(A)**, *Triatoma dimidiata* **(B)**, *Triatoma rubrovaria* **(C)**, *Triatoma vitticeps* **(D)**, *Panstrongylus megistus* **(E)**, *Rhodnius neglectus* **(F)**, and *Rhodnius prolixus* **(G)**. The values are mean and SEM obtained from groups of 15 insects. Asterisks indicate significant differences compared to day 0. Statistics: Unpaired t-test (*T. infestans*, *P. megistus*, *R. neglectus* and *R. prolixus*); Mann-Whitney test (*T. dimidiata*, *T. rubrovaria* and *T. vitticeps*). **p < 0.01; ****p < 0.0001.

**FIGURE 5:**
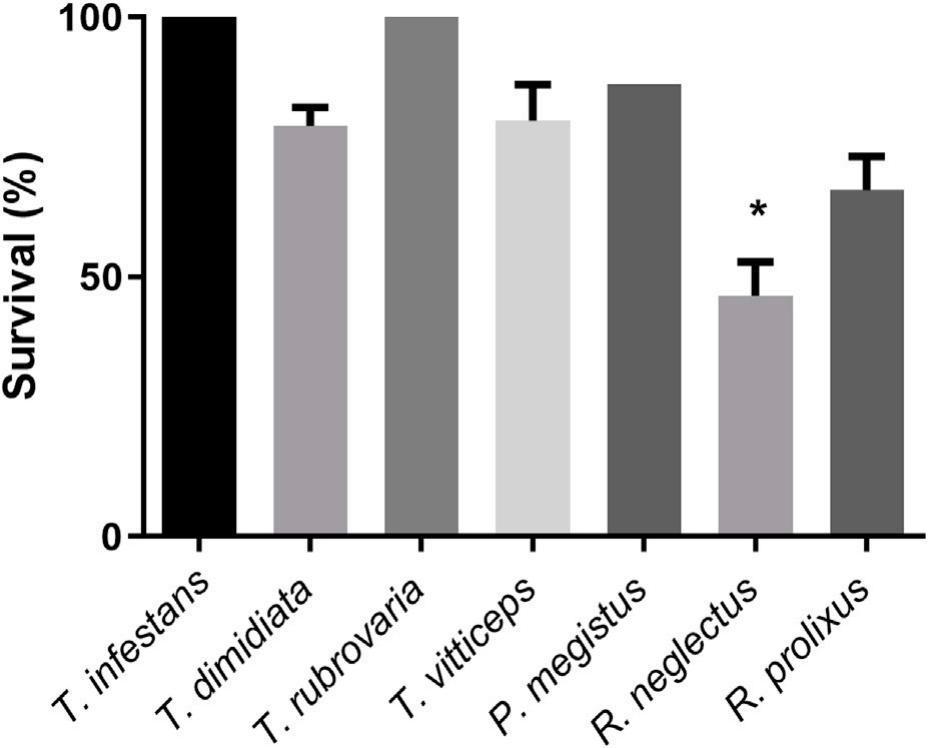
Survival of first instar nymphs of different triatomine species after 2 days of exposure to sucrose baits: *Triatoma infestans, Triatoma dimidiata, Triatoma rubrovaria, Triatoma vitticeps, Panstrongylus megistus, Rhodnius neglectus, and Rhodnius prolixus*. The values are mean and SEM obtained from groups of 15 insects. The experiment was repeated three times independently. Asterisks indicate statistical differences (Fisher’s exact test), *p* < 0.05.

Sucrose solution was offered to first instar nymphs of *R. neglectus*, *R. prolixus*, *T. dimidiata*, *T. vittticeps*, *T. rubrovaria*, *T. infestans* and *P. megistus*. At least single nymphs were nearly fully or partially engorged (Figure 3; green and yellow arrows, respectively). None of the control insects ingested water (red arrows). Whereas all *T. dimidiata* showed an increase in weight, the increases varied in the other species (Figure 4). After ingestion of low volumes, none of the first instars of *T. infestans* and *T. rubrovaria* died within 2 days (Figure 5). The mortality rates of *R. prolixus*, *T. dimidiata*, *T. vitticeps* and *P. megistus* ranged between 10% and 30%, but 50% died in the group of *R. neglectus*.

### 3.2 Effect of toxic sugar baits on *R. prolixus*

**FIGURE 6:**
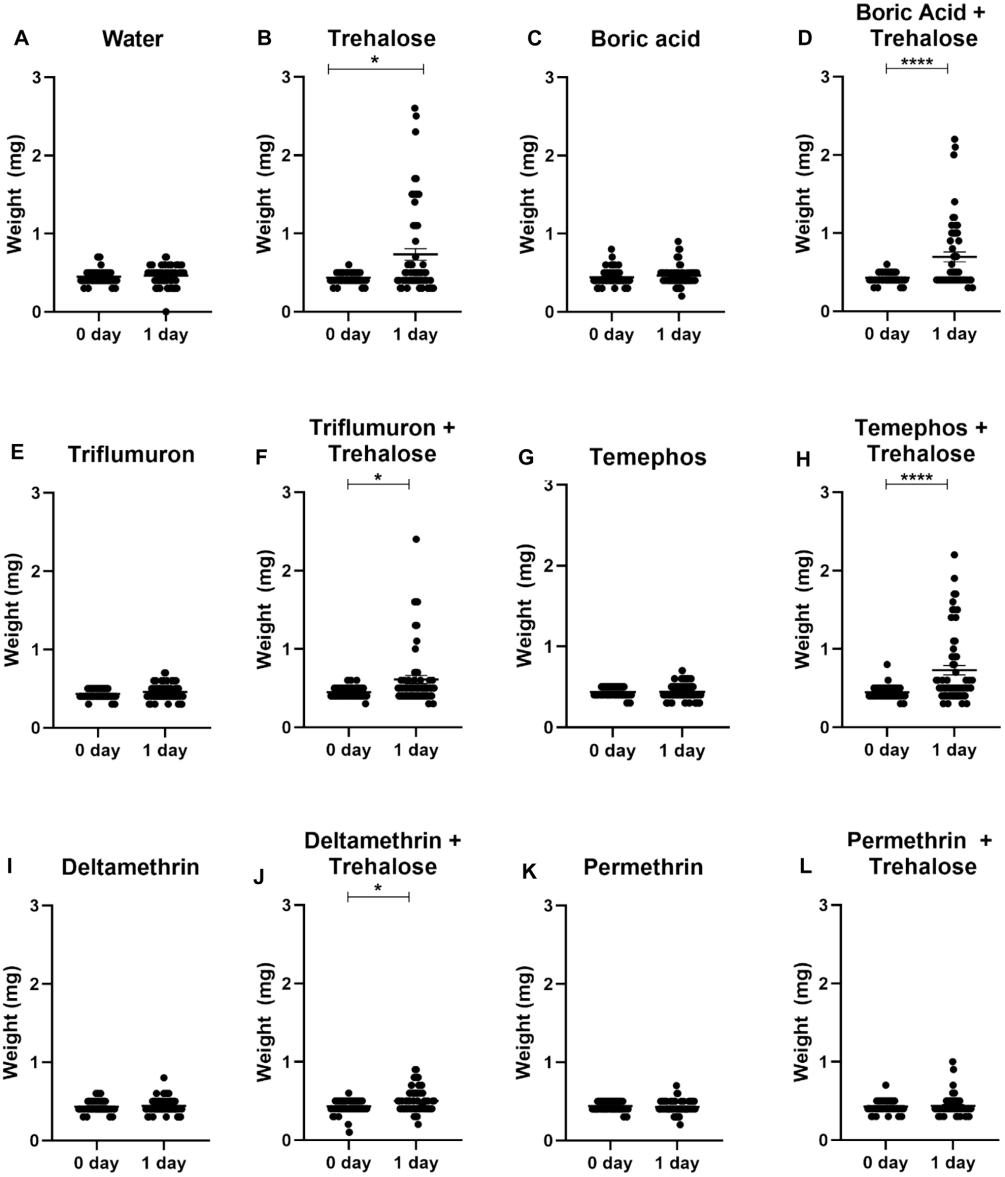
Weights of first instar nymphs of *Rhodnius prolixus* before (0 day) and after 1 day exposure to baits with water **(A)**, trehalose **(B)**, boric acid **(C)**, boric acid + trehalose **(D)**, triflumuron **(E)**, triflumuron + trehalose **(F)**, temephos **(G)**, temephos + trehalose **(H)**, deltamethrin **(I)**, deltamethrin + trehalose **(J)**, permethrin **(K)**, permethrin + trehalose **(L)**. The experiment was repeated three times independently, with 20 insects in each group. Asterisks indicate significant differences compared to day 0. Statistics: Unpaired t-test (triflumuron, temephos and permethrin); Mann-Whitney test (water, trehalose, acid boric, acid boric + trehalose, triflumuron + trehalose, temephos + trehalose, deltamethrin, deltamethrin + trehalose and permethrin + trehalose). *p < 0.05; ****p < 0.0001.

**FIGURE 7:**
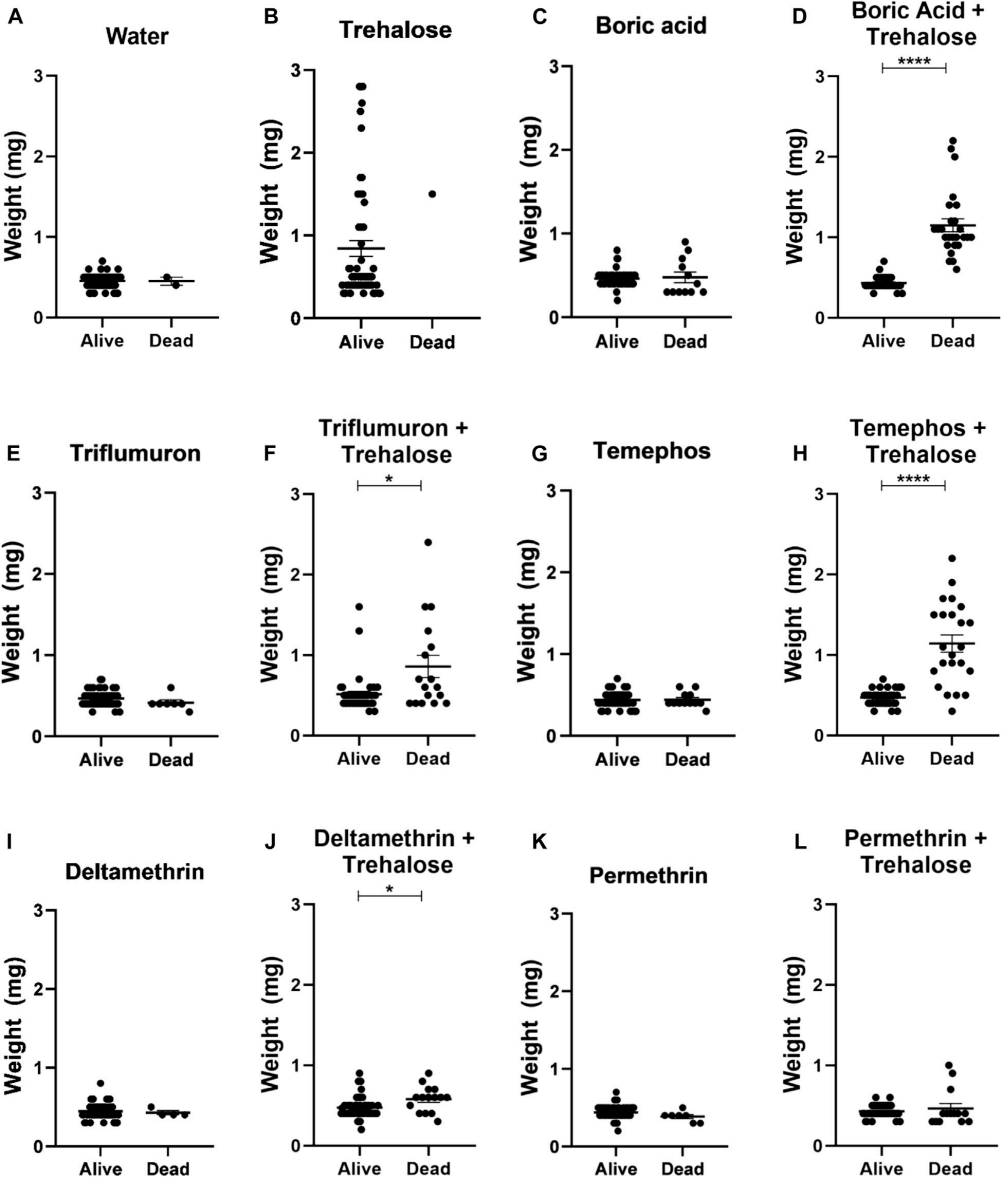
Weights of live and dead first instar *Rhodnius prolixus* nymphs after 1 day of exposure to baits with water **(A)**, trehalose **(B)**, boric acid **(C)**, boric acid + trehalose **(D)**, triflumuron **(E)**, triflumuron + trehalose **(F)**, temephos **(G)**, temephos + trehalose **(H)**, deltamethrin **(I)**, deltamethrin + trehalose **(J)**, permethrin **(K)**, permethrin + trehalose **(L)**. The experiment was repeated three times independently, with 20 samples in each group. Asterisks indicate significant differences compared to day 0. Statistics: Unpaired t-test (triflumuron, temephos and temephos + trehalose); Mann-Whitney test (water, trehalose, acid boric, acid boric + trehalose, triflumuron + trehalose, deltamethrin, deltamethrin + trehalose and permethrin + trehalose). *p < 0.05; ****p < 0.0001

Nymphs exposed to trehalose (control), boric acid + trehalose, triflumuron + trehalose, temephos + trehalose and deltamethrin + trehalose significantly increased their weight after 24 h of exposure to the different treatments, indicating that the insects ingested the insecticides in the presence of sugar (Figure 6). On the other hand, in nymphs exposed to water (control) and the insecticides without trehalose rarely the weight increased. This also occurred in the exposure to permethrin + trehalose. Since only two nymphs had ingested a small volume, the taste of pyrethroids might act against an ingestion. Analyzing the individual weights of live and dead insects after 24 h of exposure, in the insecticide + sugar combinations, the dead insects were those that were completely or partially engorged (Figure 7). Therefore, sugar had a phagostimulant effect on triatomines, and increased the effect of the toxic sugar baits in *R. prolixus* nymphs.

**FIGURE 8:**
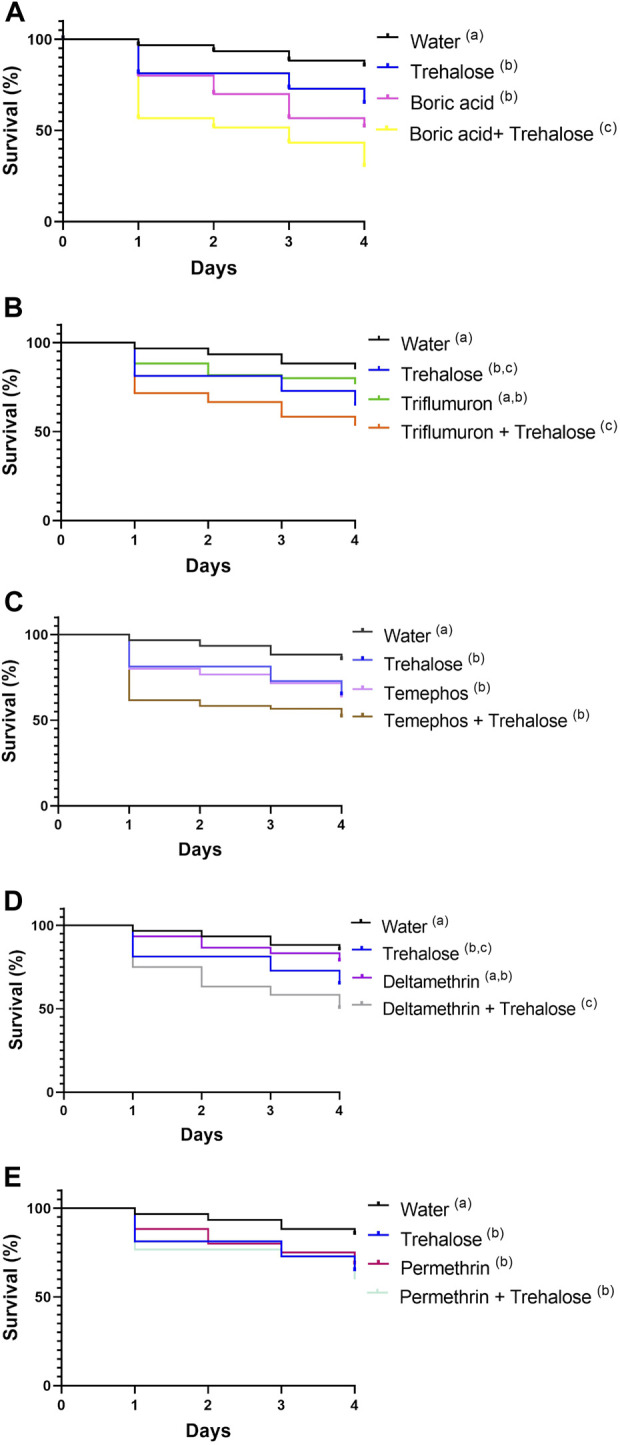
Survival of first instar nymphs of *Rhodnius prolixus* during exposure to baits containing: boric acid, boric acid + trehalose **(A)**, triflumuron, triflumuron + trehalose **(B)**, temephos, temephos + trehalose **(C)**, deltamethrin, deltamethrin + trehalose **(D)**, permethrin, permethrin + trehalose **(E)**; water, trehalose (controls). All experiments were performed simultaneously with the same control groups. The experiment was repeated three times independently, with 20 insects in each group. Different letters denote curves that are significantly different by log-rank analysis (Mantel-Cox, *p* < 0.05).

The presence of sugar with the boric acid insecticide resulted in a significant impact on insect survival, proved to be quite toxic for this species (Figure 8A; Supplementary Table S1A). Nymphs exposed to water had a survival rate of 85% after 4 days, trehalose 64.5%, boric acid 51.6% and boric acid + trehalose 30%.

Insects exposed to triflumuron had a survival after 4 days of 76.6% and triflumuron + trehalose 53.3% (Figure 8B; Supplementary Table S1B). These conditions were significantly different to each other (Supplementary Table S1B). Nymphs exposed to temephos after 4 days showed survival of 63.3% and temephos + trehalose 51.6% (Figure 8C), and both groups showed significant reduction in survival when compared to water (Supplementary Table S1C). Insects exposed to deltamethrin showed survival after 4 days of 78.3% and deltamethrin + trehalose of 50% (Figure 8D). The survival rate of the group treated with deltamethrin + trehalose was significantly lower when compared to deltamethrin or water groups (Supplementary Table S1D). Nymphs exposed to permethrin after 4 days had a survival of 68.3% and permethrin + trehalose 60% (Figure 8E). The survival rates of both groups above were reduced significantly when compared to the control group with water (Supplementary Table S1E).

## 4 Discussion

The study of the feeding behavior in kissing bugs was crucial to better understand its basic biology and, thus, develop new control strategies to reduce the population of Chagas disease vectors (Oliveira and Genta, 2021). In this study, we tested several sugars, in different species of triatomines, to characterize the sugary solutions intake and physiological effects on these insects. Additionally, we tested sugar baits as an insecticide delivery strategy for triatomine bugs. All instars of *R. prolixus* (except adults) ingested sucrose in laboratory conditions. No water engorgement was observed in *R. prolixus* nymphs and adults, demonstrating that insects are not significantly attracted to water. Interestingly, sugar intake is more frequent in first instar nymphs. This probably happened because adult insects had greater energy reserves and were not in the same stage of fasting and malnutrition (Collier et al., 1981). The mortality in triatomines after exposure to sucrose was directly related to their specific age, as first instar nymphs had lower survival. Nymphs engorged more frequently when they were exposed to 10% sucrose and not to water. However, mortality was higher at this concentration. In other concentrations of sucrose, insects showed no avidity and, consequently, do not become engorged and do not die (data not shown). First instar nymphs of *R. prolixus* exposed to *S. lycopersicum* (cherry tomatoes) ingested plant tissues, and that improved the fitness of the insect (Díaz-Albiter et al., 2016). An increase in the amount of blood ingested and urine excreted was observed, such as decreased mortality, increased longevity, and reduced weight loss caused by desiccation. Taken together with our data, the physiological gain in insects exposed to tomato probably was not due to ingested sucrose, but perhaps to other sugars or other compounds of the secondary metabolism of the plant, which had beneficial effects for *R. prolixus*. It is clear that sugary feeding was not enough as an exclusive food source for triatomines, because these insects did not complete their biological cycle without a blood meal (Garcia and Azambuja, 1985). It was not clear if sugary feeding has a nutritional role in the physiology of kissing bugs in nature. However, it might be possible that during a period of food restriction, these insects could alternatively feed on vegetables, and sugary food could somehow keep the insect alive for longer periods of fasting. In this respect, da Lage et al. (2024) recently reported the presence of plant DNA in field triatome samples, and the presence of amylase in *R. prolixus* genome.

As mentioned before, triatomines had different food sources in the laboratory. Nymphs ingested the hemolymph of cockroaches, and in the absence of vertebrates, haemolymphagy could be an alternative food source as a means of survival for the insect (Lorosa et al., 2000). This is coherent with the lack of toxicity observed for trehalose in our experiments. However, this behavior is restricted to some triatomine species (Durán et al., 2016).

When we performed a screening with different sugars, there was an indiscriminate intake, but with different physiological outcomes. The toxicity of some sugars, especially polysaccharides, was not conclusively assessed, due to the lack of ingestion. Triatomines that fed on trehalose showed a high engorgement rate and none died. Probably the effects of ingested trehalose were more associated with haemolymphagy rather than phytophagy. Therefore, there was a possibility that the insects were adapted to ingest this sugar. In summary, the sugary feeding in triatomines was completely nonspecific, with no discrimination between different sugars, and the detection of the sugar solution seemed to occur after initial contact. However, in our experiments there was no choice between sugars, so the insect had only the options between feeding or starving. Other experimental designs must be used to assess particular feeding preferences.

The polysaccharides that were not ingested as starch, carboxymethylcellulose, laminarin, pectin, and xylan were probably sugars with low sweet taste. Triatomines preferred sweeter sugars, such as sucrose. Another factor that possibly contributed to the low ingestion was the higher viscosity of the solutions, which may had decreased the palatability and, consequently, the attractivity to the nymphs.

All the seven tested triatomine species ingested sugar solutions and significantly increased their weight, and showed a phagostimulating effect, but engorgement varied according to the species. Of note, when these insects were exposed to water or saline solutions, we did not observe engorgement (data not shown). Engorgement was previously seen only when the insect feeds on blood, ATP, or 2,3-diphosphoglycerate. ATP was widely known in the literature as a chemical phagostimulant in *R. prolixus* (Friend and Smith, 1971; Friend and Smith, 1982), as well as 2,3-diphosphoglycerate (Macarini, 1983). Therefore, it was the first time that sugars were reported to be a phagostimulants for triatomines.

Accordingly, 1st instar nymphs of *R. prolixus* ingested different combinations of insecticides + sugar and the presence of sugar in the insecticide bait significantly reduced insect survival. Remarkably, among all the insecticides tested, boric acid was highly effective, since we observed a high rate of engorgement and a low survival rate when compared to the controls. The results were in accordance with several studies of toxic sugar baits where boric acid has been shown to be effective against various dipterans (Naranjo et al., 2013; Bhami and Das, 2015). Toxic sugar baits may be an interesting new tool for the control of triatomines. Sugar feeding resulted in ingestion of the insecticide by the triatomine, which meaned a route of action that is completely different to the current insecticides available for triatomine control, that work by residual contact with treated surfaces. This might have implications for exploring new insecticide mechanisms, as well as making the surviving of refractory individuals more difficult.

An important consequence of this new delivery system for triatomines was the use of lower concentrations of insecticides, when we compared to other strategies. For example, a bait with hexachlorocyclohexane 1% (v/v) that explored the water drinking behavior of triatomines was proposed (Lima et al., 1992). For comparison, we observed similar results using 0.01% boric acid. For the pyrethroids tested, it was difficult to make a direct comparison, because the known toxicities for triatomines were reported in contact experiments, after the treatment of surfaces. However, a treated paper with 25 cm^2^ contained 20 µg deltamethrin or 46 µg cypermethrin at 50% lethal concentration (Alzogaray and Zerba, 2001), an amount which was in the same range of the sugar baits presented here, with 15 µg deltamethrin or 60 µg permethrin. Nevertheless, it was clear that the environmental impact of a sugar bait was lower than a treated surface, because ingestion was a more targeted delivery mode than contact. From the insecticides tested, Triflumuron allowed the best comparison between different scenarios. *Rhodnius prolixus* was fed with blood containing 4.8 g/L Triflumuron, which is 133 times higher than the 0.036 g/L dose used in our experiments (Henriques et al., 2016). It was not clear, however, if the high toxicity observed in some of our experiments was a result of a special sensitivity of the sugar-fed gut to the insecticides, or sublethal effects of the sugars ingested. Additionally, sugar baits could be explored to the delivery of anti-parasitic compounds or microorganisms with anti-*T.cruzi* activity, in a paratransgenesis approach that was explored before by other groups, where the delivery of the microorganisms was an important limiting factor (Hurwitz et al., 2011).

In conclusion, sugar feeding was a widespread behavior in triatomines, with different physiological outcomes. *Rhodnius prolixus* actively ingested different monosaccharides and disaccharides, but this behavior was observed only in nymphs. Some of the sugars had strong toxic effects on *R. prolixus*, especially sucrose, but this effect varied in other triatomine species. However, more research is needed to explain the effects of ingestion of sugary feeding in these insects and the mechanisms of sucrose toxicity, addressing both evolutionary and physiological points of view. Besides that, different insecticides, mainly boric acid, were used in toxic sugar baits against triatomines. More detailed knowledge of the role of sugar meals in triatomines may be important for the development of new control tools against these vector insects.

## Data Availability

The original contributions presented in the study are included in the article/Supplementary Material, further inquiries can be directed to the corresponding author.
