# Erratum: Sugar feeding in triatomines: a new perspective for controlling the transmission of Chagas disease

**DOI:** 10.3389/fphys.2024.1501103

**Published:** 2024-10-16

**Authors:** Frontiers Production Office

**Affiliations:** Frontiers Media SA, Lausanne, Switzerland

**Keywords:** triatomine, sugar feeding, chagas disease, attractive toxic sugar bait (ATSB), *Rhodnius prolixus*

Due to a production error, an incorrect **editor’s** name was listed. The correct name of the editor is “Norman Arthur Ratcliffe.”

The publisher apologizes for this mistake. The original version of this article has been updated.

