# Supplementary material for: Sugar feeding in triatomines: a new perspective for controlling the transmission of Chagas disease
Source: Front Physiol. 2024 Oct 15;15:1360255. doi: 10.3389/fphys.2024.1360255 (PMC11231924; doi:10.3389/fphys.2024.1360255)
Supplement: Supplementary file 1 [file Table1.DOCX]

**Supplementary Table 1.** Results of statistical analysis of comparisons between survival curves of first instar nymphs of *Rhodnius prolixus* during exposure to baits containing different insecticides with or without trehalose, corresponding to the data presented in the Figure 8 (8A-8E). (A) Boric Acid; (B) Triflumuron; (C) Temephos; (D) Deltamethrin; (E) Permethrin. *p* values are presented in italics, and Chi-squares are in normal characters. Comparisons with significant differences have their *p* values in bold numbers.

| A | H_2_O | Trehalose | Boric Acid | Boric Acid + Trehalose |
| --- | --- | --- | --- | --- |
| H_2_O |  | ***0.0126*** | ***< 0.0001*** | ***< 0.0001*** |
| Trehalose | 6.22 |  | *0.1644* | ***0.0003*** |
| Boric Acid | 16.09 | 1.93 |  | ***0.0142*** |
| Boric Acid + Trehalose | 39.39 | 13.24 | 6.01 |  |

| B | H_2_O | Trehalose | Triflumuron | Triflumuron + Trehalose |
| --- | --- | --- | --- | --- |
| H_2_O |  | ***0.0126*** | *0.2198* | ***0.0001*** |
| Trehalose | 6.22 |  | *0.1892* | *0.1972* |
| Triflumuron | 1.51 | 1.72 |  | ***0.0073*** |
| Triflumuron + Trehalose | 14.98 | 1.66 | 7.20 |  |

| C | H_2_O | Trehalose | Temephos | Temephos + Trehalose |
| --- | --- | --- | --- | --- |
| H_2_O |  | ***0.0126*** | ***0.0059*** | ***< 0.0001*** |
| Trehalose | 6.22 |  | *0.8678* | *0.1367* |
| Temephos | 7.59 | 0.02 |  | *0.1535* |
| Temephos + Trehalose | 16.47 | 2.22 | 2.04 |  |

| D | H_2_O | Trehalose | Deltamethrin | Deltamethrin + Trehalose |
| --- | --- | --- | --- | --- |
| H_2_O |  | ***0.0126*** | *0.3340* | ***< 0.0001*** |
| Trehalose | 6.22 |  | *0.1122* | *0.1142* |
| Deltamethrin | 0.93 | 2.52 |  | ***0.0009*** |
| Deltamethrin + Trehalose | 17.66 | 2.49 | 11.03 |  |

| E | H_2_O | Trehalose | Permethrin | Permethrin + Trehalose |
| --- | --- | --- | --- | --- |
| H_2_O |  | ***0.0126*** | ***0.0281*** | ***0.0024*** |
| Trehalose | 6.22 |  | *0.6886* | *0.6780* |
| Permethrin | 4.82 | 0.16 |  | *0.3736* |
| Permethrin + Trehalose | 9.22 | 0.17 | 0.79 |  |
